# ACTUARIAL CRITERIA FOR MCI DIAGNOSIS IN ACTIVE: IMPLICATIONS OF ADJUSTMENT FOR RACE

**DOI:** 10.1093/geroni/igz038.1617

**Published:** 2019-11-08

**Authors:** Kelsey Thomas, Sarah Cook, Fred Unverzagt, Mark W Bondi, Michael Marsiske

**Affiliations:** 1 University of California, San Diego School of Medicine, San Diego, California, United States; 2 Duke University, Durham, North Carolina, United States; 3 Indiana University, Indianapolis, Indiana, United States; 4 University of California, San Diego, San Diego, California, United States; 5 University of Florida, Gainesville, Florida, United States

## Abstract

This study examined the baseline prevalence of mild cognitive impairment (MCI) in the ACTIVE study using actuarial criteria for MCI. Participants (n=2763; 26% Black) were classified as probable MCI cases if they had two observed test scores within the same cognitive domain (memory, reasoning, speed) that were >1SD below a demographically-adjusted expected score (based on the regression weights of a “robust” normal control group). Each score was adjusted using two approaches: Method 1 adjusted for age, sex, and education; Method 2 also adjusted for race. The estimated prevalence of MCI was 33.5% (n=925) in Method 1 and 32.1% (n=887) in Method 2. Adjusting for race reduced the proportion of Black participants classified as probable MCI from 42.3% to 34.9%. Future work will examine whether adjustment for social determinants of health (e.g., education quality, neighborhood/healthcare access) might further improve the utility of this classification method in diverse samples.

